# An exploratory feasibility study of a novel portable mainstream capnograph in a prehospital environment

**DOI:** 10.1186/s12245-026-01312-z

**Published:** 2026-07-21

**Authors:** Niccolò Pedrotti, Anders Larsson, Malin Jonsson Fagerlund, David Smekal

**Affiliations:** 1 Department for Anesthesia and Intensive Care, Uppsala University Hospital, Uppsala, Sweden; 2 Department of Surgical Sciences, Section for Anesthesiology & Intensive Care, Uppsala University, Uppsala, Sweden; 3 Department of Ambulance and Emergency Care, Uppsala University Hospital, Uppsala, Sweden; 4 Department of Medical Sciences, Uppsala University, Uppsala, Sweden; 5 Perioperative Medicine and Intensive Care, Karolinska University Hospital, Stockholm, Sweden; 6 Section of Anesthesiology and Intensive Care Medicine, Department of Physiology and Pharmacology, Karolinska Institutet, Stockholm, Sweden

**Keywords:** Capnography, Mainstream, Monitoring, Ambulance, EMS, Prehospital

## Abstract

**Background:**

Monitoring end-tidal carbon dioxide (EtCO_2_) and respiratory rate (RR) is a non-invasive and reliable, continuous breathing assessment method in intubated and spontaneously breathing patients. The prehospital spreading of this technique in awake patients has been limited by mainstream capnographs´ dimensions and costs. Yet the potential prehospital applications of a wearable mainstream capnograph are many. This study aims to assess the ability to measure EtCO_2_ of the novel mainstream *MARIE* capnograph in normal ambulance transport conditions.

**Methods:**

A single-centre, observational, exploratory feasibility study conducted in Region Uppsala between December 2024 and January 2025. First 20 healthy adult volunteers were transported approximately 10 min by ambulance, having EtCO_2_ and RR continuously measured by the mainstream device. Three measurements were performed (in the beginning, in the middle and at the end of the transport). A fourth value was noted on arrival in the Emergency Department (ED) with a reference capnograph for comparison with the third value by the mainstream device. The same measurement procedure was then repeated on 30 clinically stable adult patients transported by ambulance to the ED.

**Results:**

A total of 20 volunteers and 29 patients were included in the final analysis. The comparison between the mainstream and reference capnograph showed that median EtCO_2_ values were 4.6 [4.1–5.2] vs. 4.9 [4.4–5.2] kPa respectively (*p* = 0.35). Bland Altman analysis showed a mean difference between the mainstream capnograph and reference device of 0.16 kPa (95% CI -0.07-0.41), with moderate correlation between measurements. The median values of breaths per minute were 17 [14–21] vs. 19 [15–22] respectively (*p* < 0.01).

**Conclusion:**

This study suggests that the mainstream capnograph *MARIE* is able to measure credible EtCO_2_ values in standard ambulance transport conditions.

**Clinical trial registration:**

ClinicalTrials.gov, TRN NCT06905613, Registration date 16th December 2024.

**Supplementary Information:**

The online version contains supplementary material available at 10.1186/s12245-026-01312-z.

## Introduction

Capnography is a non-invasive method to continuously monitor breathing by measuring end-tidal carbon dioxide (EtCO_2_) and creating a waveform. Often combined with respiratory rate (RR), capnography is today basic requirement during anaesthesia and intensive care to assess ventilation in patients requiring assisted or controlled ventilation under sedation or general anaesthesia [1–4]. In spontaneously breathing healthy subjects, EtCO_2_ provides reliable surrogate values for the partial CO_2_ tension in arterial blood [5, 6]. The same relationship is however not seen in patients with lung pathologies such as chronic obstructive pulmonary disease (COPD) or immature lungs [7, 8], in these groups correct EtCO_2_ waveform evaluation is crucial [9]. Several reviews have recommended the use of capnography in critically ill, mechanically ventilated patients, during airway management, and during cardiopulmonary resuscitation (CPR), because capnography provides valuable information about gas exchange and dead space in acute lung injury, confirms correct endotracheal intubation, and serves as a surrogate indicator of circulatory status [9–12]. The consensus for routine use of capnography during sedation, anaesthesia and CPR is also supported by many international guidelines [13–19]. Capnography in prehospital emergency care has mainly been used as a tool to guide and optimize CPR, for confirmation of correct position of the endotracheal tube and during transfer of intubated, critically ill patients [11, 12]. This technology has also been studied in the diagnosis of pulmonary embolism, but its adoption in the prehospital setting among spontaneously breathing patients has been limited by the lack of affordable, reliable instruments [20, 21]. Capnographs intended for spontaneously breathing patients traditionally use side-stream technology with a suction pump that draws a small flow of sample gas from the patient to the monitor where the gas analysis sensor is located. Side-stream technology implies the use of relatively long tubing system (susceptible to kinking and obstruction), a water trap and a suctioning pump. Mainstream capnographs have the gas analysis sensors close to the patient, producing an undistorted waveform even at high RR but they are often not perfectly designed to carry at the patients’ side in the prehospital setting. Mainstream technology is considered superior for confirmation of proper endotracheal intubation [22], since the gas analysis sensor is positioned directly within the path of gas flow, as opposed to the side-stream sensor located off the main path of flow in the airway circuit. This superiority has been seen also among postoperative non-intubated patients and in ICU patients with non-invasive ventilation (NIV) following extubation [23, 24]. There are many potential applications of a small, wearable, battery-driven, mainstream capnograph in a prehospital emergency care environment where access to injured patients is both challenging and crucial.

The primary objective of this study was to assess whether a novel, portable, mainstream capnograph called *MARIE* could measure and show credible EtCO_2_ values under standard ambulance transport conditions. Secondary objectives were to measure eventual differences in EtCO_2_ values between *MARIE* and side-stream reference devices and to assess the ability to measure RR of the mainstream capnograph.

## Methods

### Study design

This is a single-centre, observational, prospective, exploratory feasibility study. The “*STARD”* reporting guideline was used to guide the reporting of our study, where applicable [25].

### Setting

A novel, mainstream capnograph was tested in ambulances in Region Uppsala, Sweden. This region has an area of 8209 km^2^, around 400.000 inhabitants and is served by one university hospital and one regional hospital. The Emergency Medical Service (EMS) system in this region has four ambulance stations and one single regional Emergency Medical Dispatch (EMD). Every ambulance is staffed by either two nurses or one nurse and an emergency medical technician. In our setting, as in most parts of Sweden, ambulance capnography is only used during CPR, while ventilation in spontaneously breathing patients is mostly assessed through clinical assessment and rarely through electrocardiogram-based RR recorders.

### Capnography devices and measurements

**Fig. 1:**
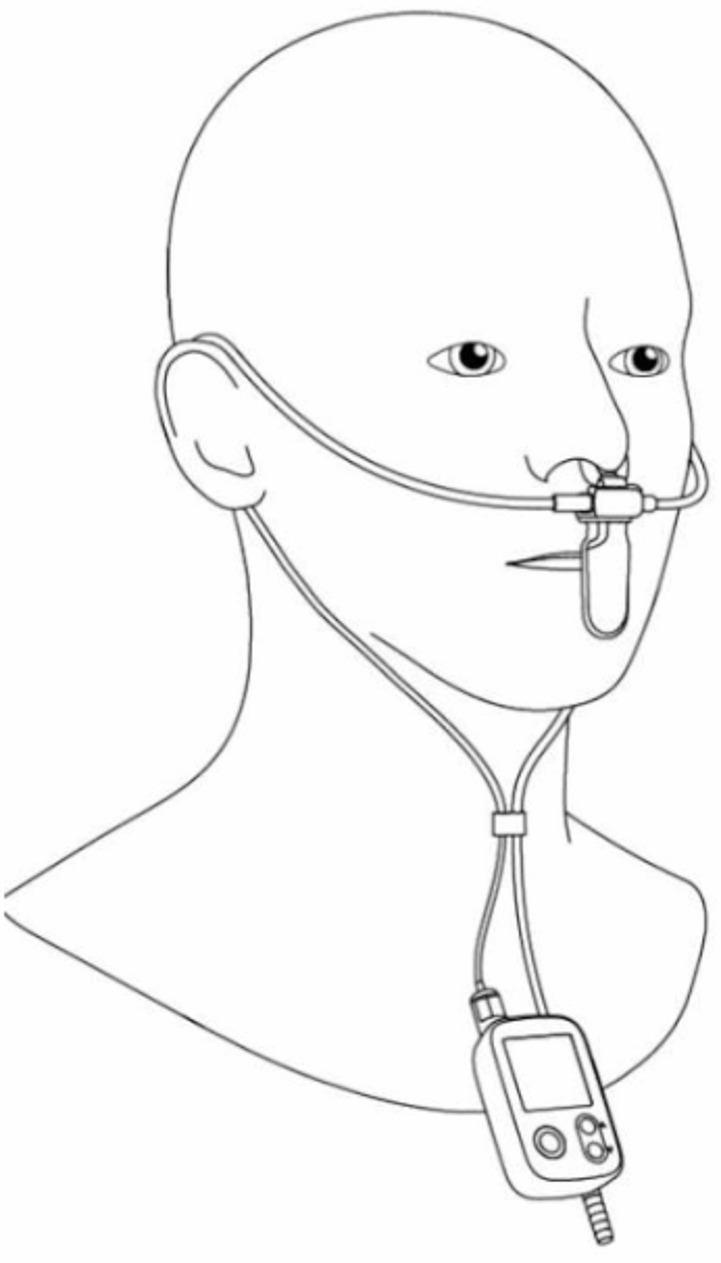
The mainstream capnograph, *MARIE*

The mainstream capnograph assessed in the study was called *MARIE*, third prototype version: *P3* (Fig. 1). It has three main components: a disposable nasal cannula, a cabled mainstream infra-red sensor and a battery-driven monitor. EtCO_2_ measurements, a capnography waveform and RR values appear directly on the monitor screen without calibration delays. Extra oxygen can be delivered through the device but this function was not tested in this trial. The monitor´s dimensions are 70 × 43 × 20 mm and the total weight is 57.5 g (56 g for the monitor + 1.5 g for the sensor cable). The accuracy of the *MARIE* device regarding EtCO_2_ is stated by the producer to be ± 0.5 kPa (calculated assuming an EtCO_2_ value of 5 kPa) and ± 1 breaths per minute regarding RR. Measurements performed with this mainstream capnograph are labelled with the letter “M”.

Reference EtCO_2_ measurements were carried out with capnographs placed in the ED. These measures were performed in the ED’s ambulance garage as soon as the patient was leaving the ambulance in order to minimize the time between the last measure performed with the mainstream capnograph and the reference capnograph. The devices used were either *Medtronic Capnostream 35* or *Philips IntelliVue X3*. Both devices measure EtCO_2_ through a disposable nasal cannula using side-stream technology, show a capnography waveform and RR values on their screens and have a weight of 1 kg vs. 1.4 kg respectively. Both devices have an accuracy of ± 0.26 kPa for EtCO_2_ (calculated assuming an EtCO_2_ value of 5 kPa) and ± 1 breaths per minute for RR. They share the same underlying technology, the *X3* being an evolution of the *Capnostream 35*. Measurements obtained with these side-stream capnographs are denoted by the letter “S”.

To reach the primary objective of the study, EtCO_2_ values obtained with the mainstream capnograph should fall within the normal physiological range of 4.5–6.0 kPa. A secondary objective was that the difference in EtCO_2_ between the mainstream device (M3) and the side-stream reference device (S1) should remain within the predefined acceptance interval based on accuracy specifications. At 5 kPa, this acceptance interval was 0.76 kPa, requiring the difference between M3 and S1 to be less than 0.8 kPa. This threshold is consistent with the minimum accuracy requirement for EtCO_2_ specified in the technical standard for respiratory gas monitors (± 0.43 kPa + 8% of the measured gas level = ± 0.83 kPa at 5 kPa).

### Participants and study protocol

**Fig. 2:**
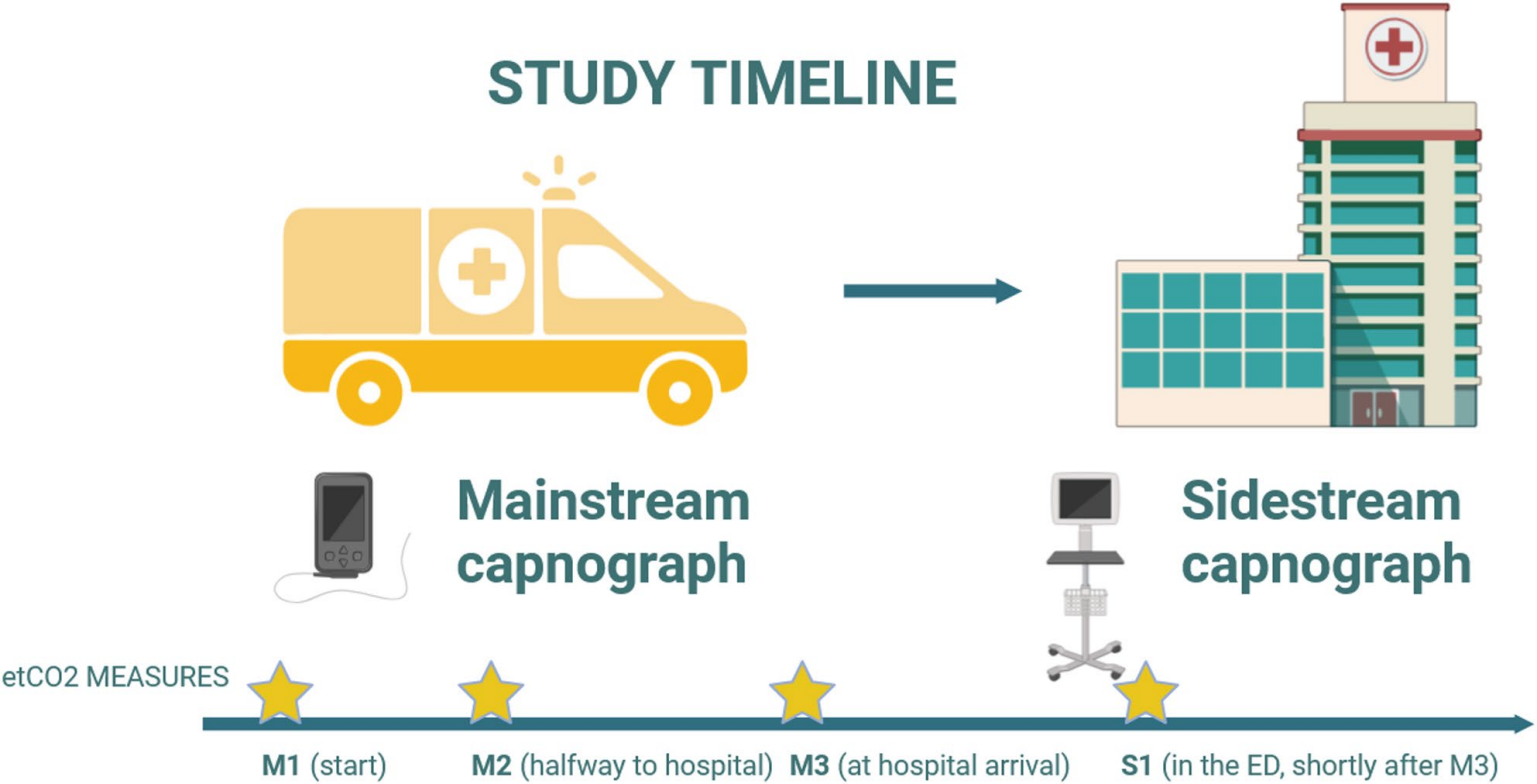
Study timeline

In the first part of the study, after signing an informed consent form, twenty healthy adult volunteers were transported by ambulance between Uppsala ambulance station and Uppsala University Hospital ED. End-tidal CO_2_ and RR were measured continuously by the mainstream capnograph (M), and the values were noted at the beginning, in the middle and at the end of the transport at arrival to the ED (timing labels: M1, M2 and M3 respectively) on a paper CRF. Directly after arrival at the ED the nasal canula was shifted to another canula connected to a reference side-stream capnograph (S) and after about 20 s an EtCO_2_ value and a RR were noted (timing label: S1) in the study CRF (Fig. 2). For logistical reasons, and to minimize the time interval between M3 and S1, the same ambulance was used for all transports of healthy volunteers. All measurements in this group were performed on the same day, in this cohort, the healthy volunteers served as their own case-controls, being measured with two different devices. As no adverse events occurred during this first part of the study, we proceeded to the second part. Thirty, consecutive, adult non-critical patients transported to ED by ambulance in Region Uppsala were included. To minimize interference with the measurements, and due to patient safety concerns raised by the ethical authorities monitoring the study, patients were excluded if they needed supplemental oxygen, if their condition justified a transport with “*lights and sirens*” and in case of known pregnancy. After providing informed consent, patients were connected to the mainstream capnograph. EtCO_2_ and RR were recorded at the beginning and midpoint of transport (M1 and M2), and a final measurement (M3) was obtained with the mainstream capnograph just before the patient was removed from the ambulance. The reference measurement (S1) was then performed with the side-stream capnograph immediately afterward, in the ambulance garage directly connected to the ED. All measurements in the patient group were performed at the bedside by the EMS personnel simultaneously providing patient care; all EMS personnel were instructed to give full priority to patient care. Unexpected adverse events, capnograph malfunction, and unplanned deviations from the study protocol were also recorded in the case report form (CRF) and reported as part of the results.

### Analysis

To detect a clinically relevant deviation with sufficient statistical power, the required sample size was calculated to be at least 30 subjects. To minimize risk to patients, the study began with 20 healthy volunteers; combined with the subsequent patient cohort, this exceeded the total of 30 subjects required for adequate statistical power. Characteristics were described as number (percentage) for categorical variables and mean (± standard deviation, SD) or median [interquartile range, IQR] for continuous variables as appropriate. To assess differences between normally distributed data t-tests were used and Mann-Whitney tests were used for non-normally distributed data. Deming regression was used to assess correlation between the new mainstream capnograph and reference capnograph, while Bland-Altman plot was used to assess eventual systematic bias between the two methods. A pre-defined significance level of *p* < 0.05 was used in all cases. Data analysis was performed with “*Excel”* (Microsoft Excel for Microsoft 365, v2108) and “*R”* version 4.3.2 [26].

## Results

### Demographics

**Fig. 3:**
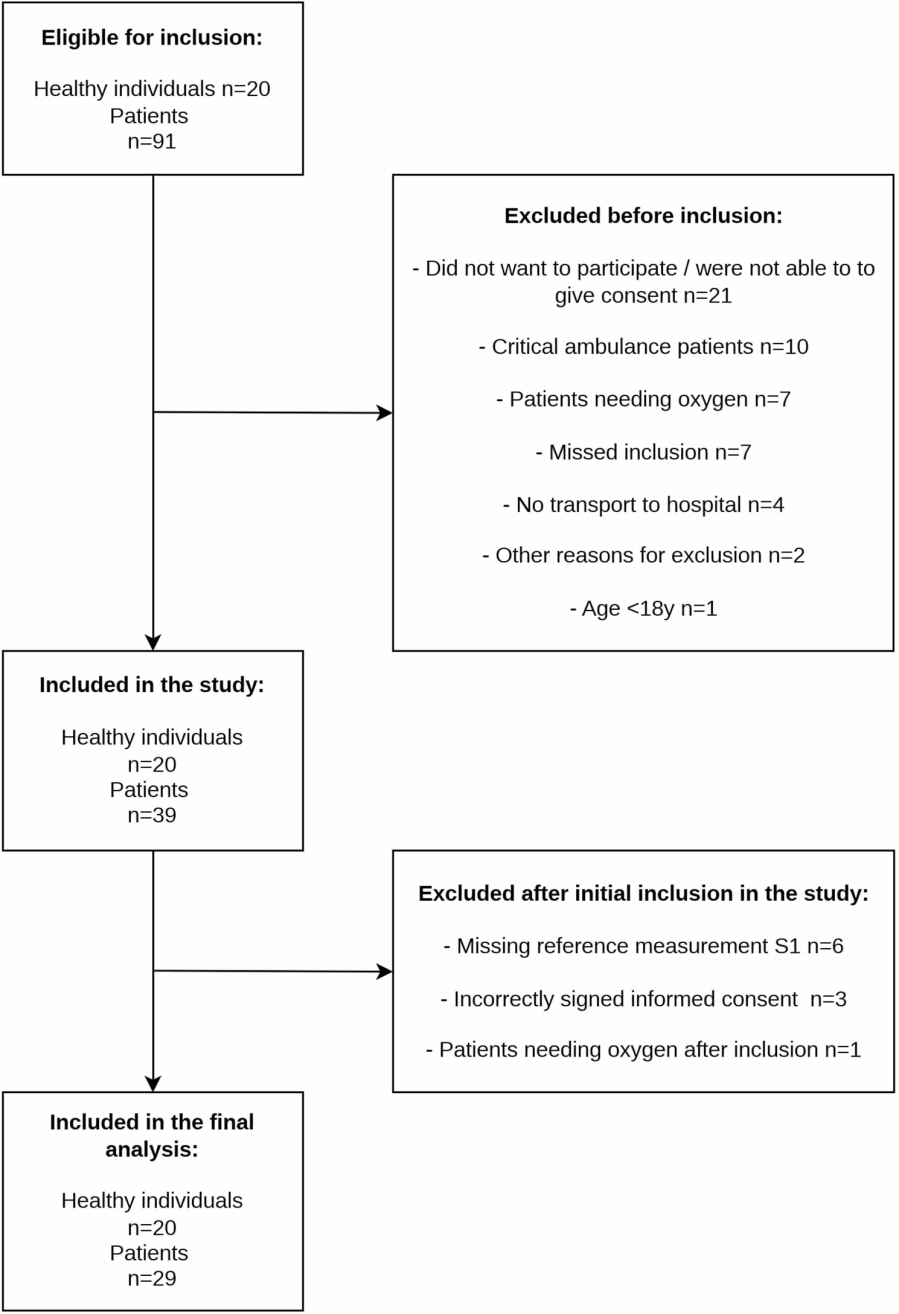
Study flow diagram

Between December 18th 2024 and January 25th 2025, 20 healthy subjects and 91 patients were considered eligible for inclusion, of these, all 20 healthy subjects and 29 patients were included in the final analysis of the study. Participants with missing data and/or missing signed informed consent were excluded from the study (Fig. 3). The most common reasons for the ambulance dispatch were chest pain, fainting, dizziness, trauma and decreased general condition (Table 1 of the supplemental material). Demographic and baseline data of study population were as presented in Table 1. Among the subjects with a comorbidity, 5 patients had asthma and 3 patients had COPD.

**Table 1:** Study population demographics. 49 subjects were included. Number of patients (%), median [Q1, Q3], EtCO_2_ and RR are presented as median values of M1, M2 and M3 measurements performed with *MARIE* capnograph

Whole population *n* = 49 (100%)		Healthy volunteers *n* = 20 (41%)	Patients *n* = 29 (59%)
Female sex	22 (45%)	6 (30%)	16 (55%)
Age (years)	53 [37, 82]	41 [34, 46]	76 [49, 83]
Past medical history	12 (24%)	4 (20%)	8 (27%)
EtCO_2_ (kPa)	4.6 [4.1, 5.2]	4.8 [4.3, 4.9]	4.5 [3.9, 5.4]
Respiratory Rate (bpm)	17 [14–21]	17 [15, 22]	17 [14, 18]

### Main results: end-tidal CO_2_ values

The comparison between measurement values at M3 with the mainstream capnograph and S1 measurements with the reference capnograph showed a mean EtCO_2_ value of 4.5 (± 1.0) kPa vs. 4.7 (± 0.7) kPa respectively, *p* = 0.35. The median time in minutes between M3 and S1 was 3 [2–6] in the whole study population, while it was 2 [1–2] vs. 5 [4–7] in heathy volunteers and in the patients group respectively. When aggregating M1, M2 and M3 the median EtCO_2_ value was 4.6 [4.1–5.2] kPa and the coefficient of variability was 22%.

### Secondary analyses

**Fig. 4:**
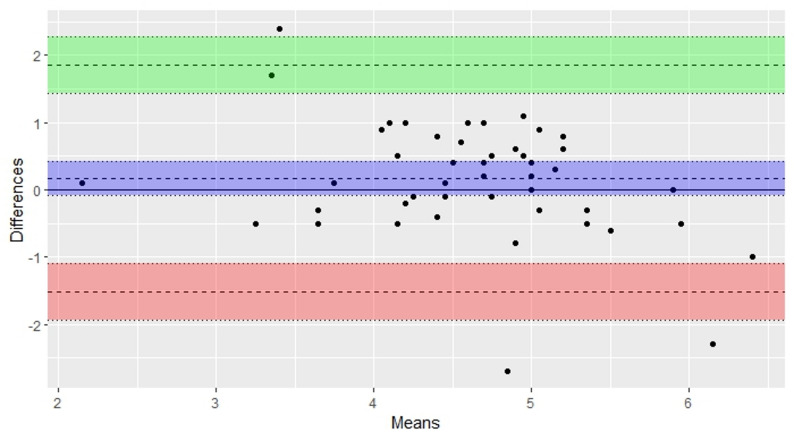
Bland-Altman plot comparing mainstream and side-stream methods (all devices *n* = 49)

**Fig. 5:**
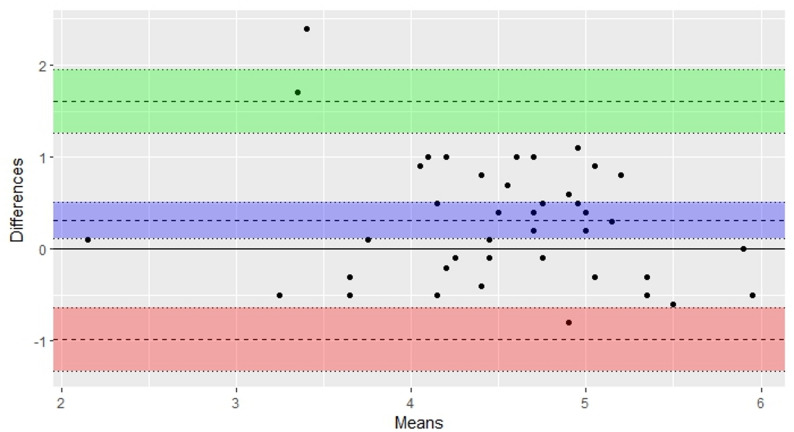
Bland-Altman plot comparing mainstream and side-stream methods (post-hoc exploratory analysis without one mainstream device with suspected drift in measures *n* = 44)

Bland-Altman analysis showed a mean difference between the mainstream capnograph and reference device of 0.16 kPa (95% CI -0.07-0.41, Fig. 4) with moderate correlation (Pearson’s *r* = 0.55, *p* = 0.17) between measurements (Fig. 1 of the supplemental material). One mainstream capnograph showed a suspected drift in the measurements with increasing values over time. A post-hoc exploratory data analysis was repeated without data from the drifting experimental capnograph (a total of 5 cases), Bland-Altman analysis showed a mean difference between the mainstream capnograph and reference device of 0.3 kPa (95% CI 0.1–0.5, Fig. 5), with still a moderate correlation (Pearson’s *r* = 0.66, *p* < 0.01) between measurements (Fig. 2 of the supplemental material).

A difference was noted in the comparison between median RR values between the values at M3 with the mainstream capnograph and the S1 measurement with reference capnograph, 17[14-21] vs. 19[15-22] breaths per minute respectively, *p* < 0.01. When aggregating M1, M2 and M3 values for RR with the mainstream capnograph the median value was 17[14-21] breaths per minute.

In all but one case, EtCO_2_ values were measured and presented during the whole transport. One mainstream device was in fact disconnected during two minutes from a patient due to motion sickness and vomiting. Neither adverse events, nor technical alarms or alarms for high/low EtCO_2_ or RR were noted.

## Discussion

Median of all EtCO_2_ values measured with the novel mainstream *MARIE* capnograph was 4.6 [4.1–5.2] kPa and no statistically significant difference was seen when comparing EtCO_2_ values measured by the mainstream and the reference side-stream capnographs, 4.5 vs. 4.7 kPa respectively (*p* = 0.35). These results suggests that the novel mainstream capnograph is able to measure credible EtCO_2_ values in standard ambulance transport conditions. Furthermore, the measured values did not statistically differ from values measured with the reference side-stream capnographs showing promising preliminary agreement. Previous studies comparing side-stream and mainstream capnography devices have reported differences in measurements ranging from 0.4 to 0.49 kPa [8]. In our study, the observed difference was smaller. However, direct comparison with cited literature is difficult because we did not measure arterial partial pressure of CO₂, which limits our ability to assess the absolute accuracy of either device.

A difference of 0.16 kPa and a moderate correlation between the two methods (*r* = 0.55, *p* = 0.17) was seen. One of the mainstream devices demonstrated a suspected drift in the measurements (higher measured EtCO_2_ values over time were noticed by the device producers in an internal analysis). A post-hoc exploratory analysis was therefore repeated without the device with the suspected drift and a difference of 0.3 kPa was seen, but the moderate correlation between the methods remained (*r* = 0.66, *p* < 0.01). This result should be interpreted with caution since it derives from a post-hoc analysis and a presumed drift. Higher EtCO_2_ values measured by the side-stream device in our group represent a surprising finding, given that S1 RR values were simultaneously higher, suggesting a higher minute ventilation. Pekdemir et al. reported a similar finding, with higher EtCO_2_ values measured by the side-stream device in subjects both with and without pulmonary pathology; however, this result warrants further investigation in future studies [8]. This feasibility study was carried out with a prototype mainstream capnograph called *MARIE P3* that might need calibration or additional software development. Further studies of a final prototype are warranted to secure that the presumed issue with drift in measurements has been eradicated. However, the EtCO_2_ difference between mainstream and side-stream capnographs was well below the pre-defined accuracy acceptance cut-off value of 0.8 kPa in both Bland-Altman analyses.

Our study showed a difference in measured RR between the mainstream capnograph and the side-stream reference device, 17 vs. 19 breaths per minute respectively, *p* < 0.01. Respiratory rate can fluctuate over short time intervals and it may as well be consciously altered by study subjects [27]. Our study design may have introduced confounding factors, such as variable timing of in-ambulance measurements (M1, M2, M3) and patient speech which likely influenced the observed RR values. However, the underlying cause of the observed lack of agreement in RR measurements between devices remains unresolved and warrants further investigation. Future studies are planned to evaluate the accuracy and reliability of the novel mainstream capnograph for RR measurement under more controlled conditions.

The mainstream capnograph showed no alarms and functioned during the whole study without the need for charging. Furthermore, both the device and the nasal cannula were easy to apply and no subject had to interrupt the study due to device discomfort. Considering these feasibility results during standard ambulance transport conditions, its small dimensions and weight, the mainstream capnograph showed potential for future prehospital implementation.

### Limitations

To the best of our knowledge, no previous research has compared mainstream and side-stream capnographs in spontaneously breathing patients in the prehospital environment. Our results might potentially contribute to the development of a portable and reliable capnograph that could be used to better monitor breathing in the largest group of patients transported by ambulance: those who are awake and spontaneously breathing. Although conducted at a single centre, the study involved all ambulance stations within Region Uppsala and two receiving hospitals, enabling rapid recruitment of both healthy volunteers and patients. To ensure adherence to Good Clinical Practice (GCP) and to maintain data integrity, an independent external monitor was appointed to oversee study conduct, verify source documentation, and ensure compliance with ethical and regulatory standards.

However, this study is not without limitations. Of 91 eligible patients, 7 were screened but not included, without documented reason. Furthermore, three patients were initially enrolled, but their data were excluded from analysis due to concerns regarding the validity of their informed consent signatures (Fig. 3). This decision was made to ensure ethical compliance and protect study integrity. Additionally, this was a pragmatic feasibility study: subjects were permitted to speak during measurements, and the timing of measurements was determined by EMS personnel based on clinical workflow. While this design minimized disruption of patient care, it may have introduced a variability affecting the quality and consistency of the measurements.

## Conclusion

Our study suggests that the portable mainstream capnograph *MARIE* can provide clinically credible end-tidal CO₂ measurements under standard ambulance transport conditions, provided that no measurement drift is present. Further studies are warranted to evaluate the final version of this device with respect to its ability to accurately measure both end-tidal CO₂ and respiratory rate even during delivery of oxygen.

## Supplementary Information

Below is the link to the electronic supplementary material.

Supplementary Material 1

Supplementary Material 2

Supplementary Material 3

Supplementary Material 4

## Data Availability

The datasets generated, used and analysed during the current study are available from the corresponding author on reasonable request.

## Abbrevations

EtCO_2_: End-tidal carbon dioxide
RR: Respiratory rate
COPD: Chronic obstructive pulmonary disease
CPR: Cardiopulmonary resuscitation
EMD: Emergency medical dispatch
EMS: Emergency medical service
ED: Emergency department
CRF: Case report form
NIV: Non-invasive ventilation
PaCO_2_: Arterial partial carbon dioxide pressure
GCP: Good Clinical Practice
