# Supplementary figures and images for: An exploratory feasibility study of a novel portable mainstream capnograph in a prehospital environment
Source: Int J Emerg Med. 2026 Jul 21;19:186. doi: 10.1186/s12245-026-01312-z (PMC13401322; doi:10.1186/s12245-026-01312-z)

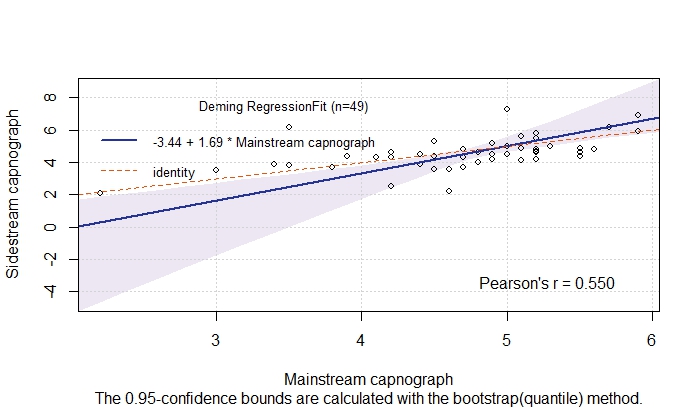

Supplement: Supplementary file 1 — Supplementary Material 1 [file 12245_2026_1312_MOESM1_ESM.jpeg]

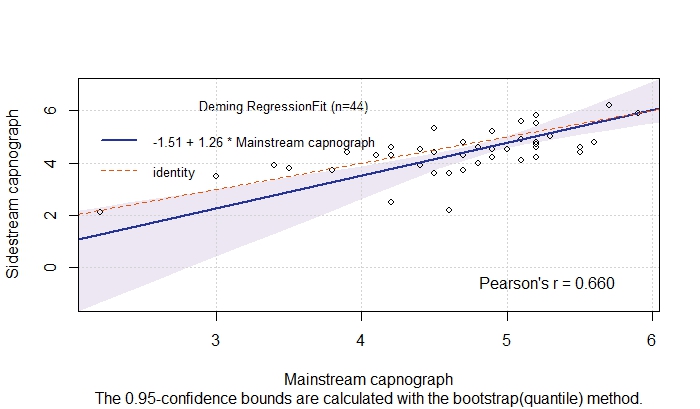

Supplement: Supplementary file 2 — Supplementary Material 2 [file 12245_2026_1312_MOESM2_ESM.jpeg]
