# Supplementary material for: An exploratory feasibility study of a novel portable mainstream capnograph in a prehospital environment
Source: Int J Emerg Med. 2026 Jul 21;19:186. doi: 10.1186/s12245-026-01312-z (PMC13401322; doi:10.1186/s12245-026-01312-z)
Supplement: Supplementary file 3 — Supplementary Material 3 [file 12245_2026_1312_MOESM3_ESM.docx]

**Table 1 supplementary material Mariestidy (editable format)**

| **Ambulance dispatch reason** | **Frequency** |
| --- | --- |
| Chest pain | 6 |
| Fainting | 4 |
| Dizziness | 3 |
| Trauma | 3 |
| Decreased general condition | 3 |
| Back pain | 2 |
| Convulsions | 2 |
| Infection | 2 |
| Abdominal/flank pain | 2 |
| Breathing difficulties | 2 |
| Urine retention | 1 |
| Stroke | 1 |
| Headache | 1 |
| Palpitations | 1 |
| Fever | 1 |
| Limb symptoms (not trauma) | 1 |

**Table 1:** Ambulance dispatch reasons with respective frequency. NB some reasons coexisted in the same patient
